# Variability of Neuronal Responses: Types and Functional Significance in Neuroplasticity and Neural Darwinism

**DOI:** 10.3389/fnhum.2016.00603

**Published:** 2016-11-25

**Authors:** Alexander V. Chervyakov, Dmitry O. Sinitsyn, Michael A. Piradov

**Affiliations:** ^1^Research Center of NeurologyMoscow, Russia; ^2^Semenov Institute of Chemical Physics, Russian Academy of SciencesMoscow, Russia

**Keywords:** variability of neuronal responses, motor evoked potentials, transcranial magnetic stimulation, neural Darwinism, degeneracy

## Abstract

**HIGHLIGHTS**

- We suggest classifying variability of neuronal responses as follows: false (associated with a lack of knowledge about the influential factors), “genuine harmful” (noise), “genuine neutral” (synonyms, repeats), and “genuine useful” (the basis of neuroplasticity and learning).
- The genuine neutral variability is considered in terms of the phenomenon of degeneracy.
- Of particular importance is the genuine useful variability that is considered as a potential basis for neuroplasticity and learning. This type of variability is considered in terms of the neural Darwinism theory.

In many cases, neural signals detected under the same external experimental conditions significantly change from trial to trial. The variability phenomenon, which complicates extraction of reproducible results and is ignored in many studies by averaging, has attracted attention of researchers in recent years. In this paper, we classify possible types of variability based on its functional significance and describe features of each type. We describe the key adaptive significance of variability at the neural network level and the degeneracy phenomenon that may be important for learning processes in connection with the principle of neuronal group selection.

Randomness is only a measure of our ignorance of the different causes involved in the production of events

**(Laplace, 1825)**

Most certainly chance is “impossible. ”There is no “chance” in Nature, wherein everything is mathematically co-ordinated and mutually related in its units. “Chance,” says Coleridge, “is but the pseudonym of God (or Nature), for those particular cases which He does not choose to subscribe openly with His sign manual.”

**(The Secret Doctrine by H. P. Blavatsky, Vol. 1)**

## Variability of neuronal responses

A fundamental problem in neuroscience is decoding of information contained in the structure and functional activity of the nervous system. Apart from its significance for the general understanding of brain functions, the problem is of increasing practical importance mainly in connection with developing the technology of brain-computer interfaces and using them to control prostheses. However, there emerges a very serious problem in the way to information decoding—variability of neuronal responses.

A stable spike frequency is detected upon registration of the membrane potential of a single neuron *in vitro*. In contrast, the spike frequency becomes irregular (variable) when activity of a similar neuron is detected *in vivo*, including cases where identical stimuli are presented (Masquelier, 2013). Similarly, spike patterns vary across trials in many cortical areas in response to sensory stimuli, as well as in the motor cortex upon making stereotyped movements. The causes and functional role of the variability are unknown.

**Figure 1:**
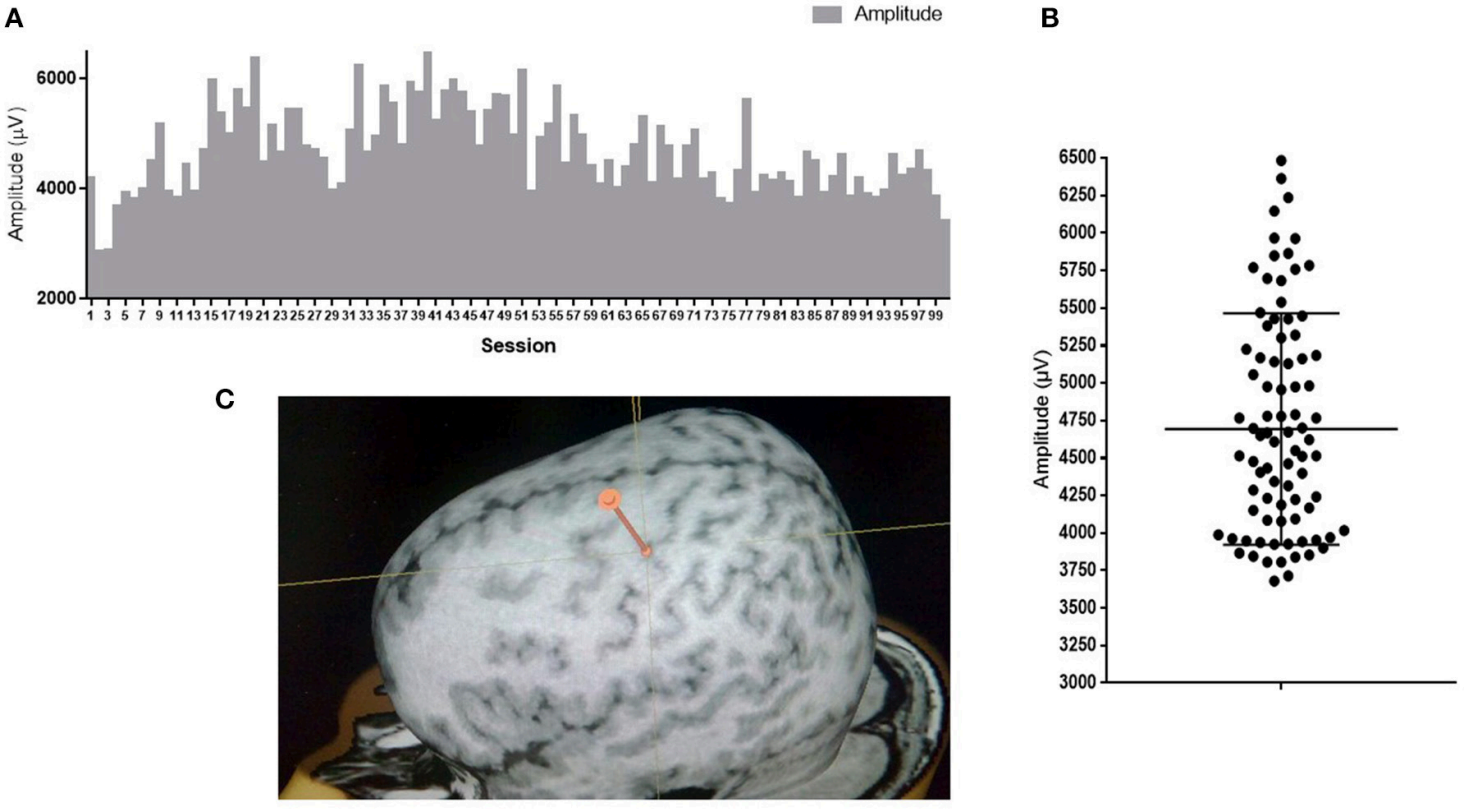
**MEP amplitudes of *m. abductor pollicis brevis* in a healthy subject; 100 TMS-stimuli in the hot spot M1 with an intensity of 110% of the response threshold**. **(A)** Amplitudes of 100 sessions one by one; **(B)** Distribution of 100 sessions amplitudes on graph. Mean amplitude ark as horizontal line. **(C)** Point of stimulation. APB hotspot.

Variability of neuronal responses can also be registered on a larger scale during detection of motor evoked potentials (MEPs) in response to stimulation of the cortex with a magnetic field pulse. Upon stimulation of the same cortical area with the same intensity using skin myographic electrodes, a MEP of a varying amplitude and latency is detected. Figure 1 shows the results of a similar experiment performed in our laboratory. The mean amplitude is 4693 μV, and the standard deviation is 766 μV, which is 16% of the mean.

Studies have shown that variability of the MEP amplitude in TMS is affected by a number of factors, including the position of a skin electrode, muscle topography (Dunnewold et al., 1998), high stimulation intensity (Pitcher et al., 2003), voluntary muscle contraction, readiness for contraction (Darling et al., 2006), gender of a subject (Pitcher et al., 2003), and presenile age (Pitcher et al., 2003). Also, factors that do not affect the variability have been identified: the size of a skin electrode (Dunnewold et al., 1998), exact positioning of a coil (navigation systems) (Jung et al., 2010), cognitive task (Kiers et al., 1993), breathing, heart rate (Amassian et al., 1990), hemisphere, and handedness.

## Explanations of variability

In modern literature, there are a number of models describing the causes of variability (Dinstein et al., 2015). Most of them are as follows. At the single cell level, response variability is determined by noise of a peripheral sensor (Schneeweis and Schnapf, 1999), stochastic nature of synaptic transmission (Ribrault et al., 2011), dynamic changes associated with neuronal adaptation (Clifford et al., 2007), and neuroplasticity (Feldman, 2009).

At the network level, the variability of neural responses in the same behavioral conditions is commonly supposed to originate from internal dynamics in the brain. Thus, Arieli et al. (1995) observed coherent ongoing activity in cat visual cortex with an amplitude almost as high as that evoked by optimal visual stimulation. They concluded that the observed activity is a result of functionally important interaction of the spontaneous activity and the evoked response. Thus, the common procedure of averaging over trials “may not be an optimal approach to study higher cognitive function, because it ignores the instantaneous state of the cortex and its influence on the individual response” (Arieli et al., 1995).

The MEP variability is explained as follows. “Related to TMS of M1, neurophysiologic parameters such as independent fluctuations in excitability of the M1 and interneurons as well as motoneurons on the spinal level (e.g., spinal desynchronization) also contribute to the variability of MEPs” (Kiers et al., 1993; Magistris et al., 1998). “Two-third of the MEP size variability is caused by the variable number of recruited α-motoneurons and approximately one-third by changing synchronization of motoneuron discharges” (Rösler et al., 2008).

## Classification of variability on the basis of functional significance

**Figure 2:**
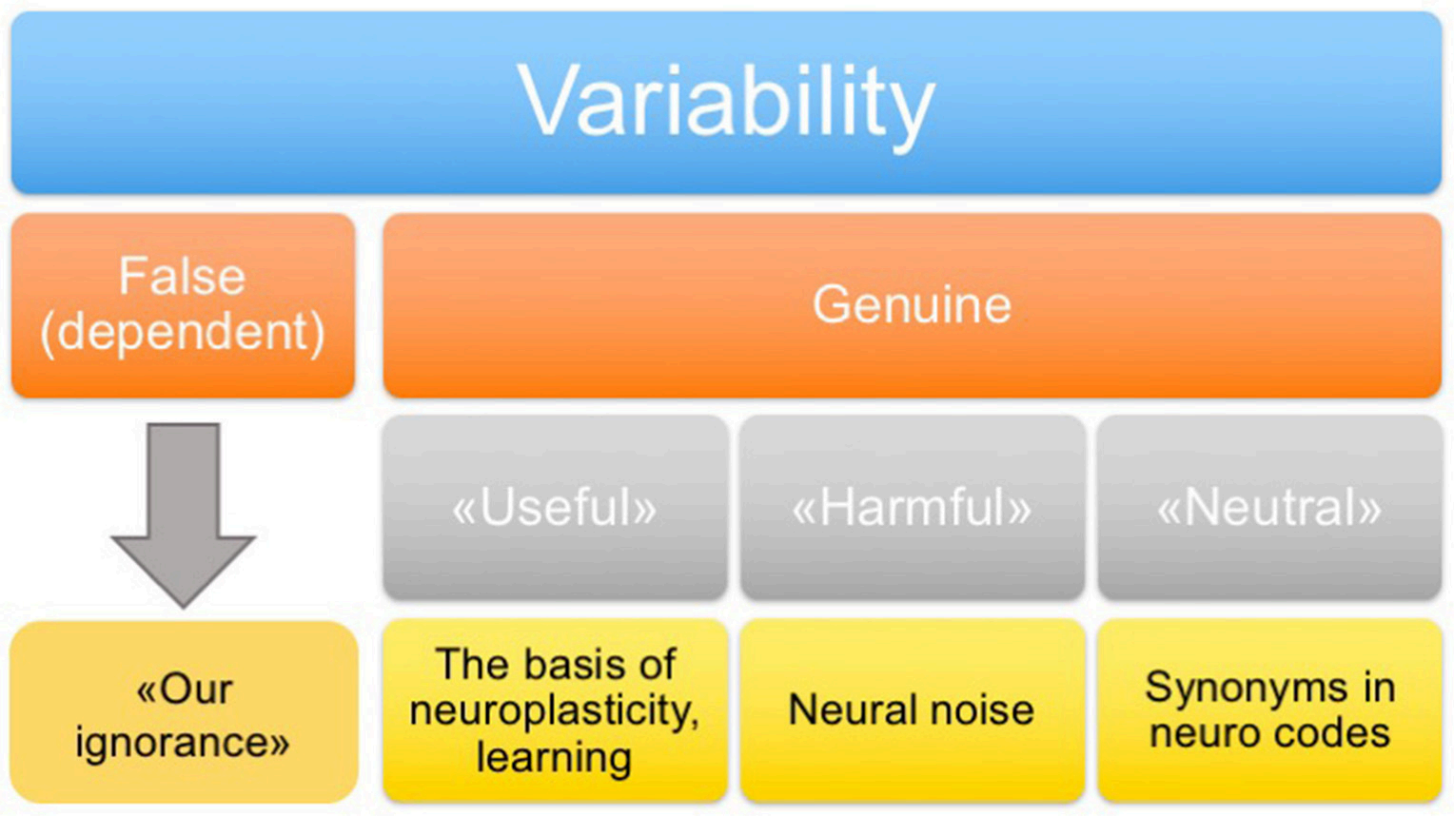
**Classification of variability**.

In addition to the classification of variability on the basis of its origin, it is also important to distinguish types of variability according to its functional role. Four types of variability may be distinguished (Figure 2): “false” (which is determined by unexplored factors), “genuine useful” (which is the basis of neuroplasticity and learning), “genuine harmful” (neuronal noise), and “genuine neutral” (a peculiarity of system functioning, including the presence of synonymous commands). In principle, the false variability may be attributed to uncontrollable factors that alter, to some extent, the cognitive task presented to the nervous system in each trial, which causes appropriate changes in the system response. In practice, a complete analysis of these factors may be extremely difficult. The harmful variability is a fundamental limitation of the precision with which the nervous system can repeat its responses under conditions imposed by a behavioral task. The useful and neutral variabilities are conceptually more complex and interesting types. They may shed light on the fundamental principles of the nervous system organization. This issue is discussed in the following sections.

## Harmful variability (noise)

A widely held view of trial-to-trial variability of neural activity, and especially of inter-spike interval patterns, considers it as random noise that limits the precision of signal representation by a neuron. Shadlen and Newsome (1998) suggested that this noise is a consequence of the maintenance of an adequate dynamic range of a neuron by balancing its excitation and inhibition, which leads to a random-walk-like pattern of spikes. This noise is supposed to be reduced at the later stages of processing by pooling signals from groups of approximately 100 neurons with similar responses.

Even if considered random, these fluctuations of neuronal activity are correlated between cells. Averbeck et al. (2006) explored the consequences of these correlations for the encoding and decoding of stimuli assuming a representation by firing rates. They found that, under certain circumstances, even week pair-wise correlations can significantly reduce the amount of information about the stimulus in a large neuronal population. The authors underline, however, that experimental assessment of the role of correlations is very hard, especially for large neuronal ensembles because of data limitations.

## Neutral variability and degeneracy

Variability of neuronal signals can occur in connection with the phenomenon of degeneracy. This term is widely used in mathematics in reference to particular cases when an object has atypical features, e.g., when a certain quantity vanishes. The meaning of the term “degeneracy” used in biology (Edelman and Gally, 2001) is close to the concept of a degenerate square matrix, i.e., a zero-determinant matrix that can produce the same vector when multiplied by different vectors. In the biological context, degeneracy is the ability of structurally different elements to perform the same function or yield the same output (Edelman and Gally, 2001). This phenomenon is distinguished from redundancy when the same function is performed by identical elements. Degeneracy can be observed in living systems at many levels of organization, from the genetic code where an amino acid is encoded by any of several possible nucleotide triplets to duplication of information by different sense organs and to a variety of ways to solve the same motor task.

Three types of degeneracy can be distinguished with regard to neural networks: parametric, dynamical, and structural. The parametric degeneracy is associated with the fact that parameters describing a particular neural network, such as ionic conductivity of neuronal membranes, can take a variety of value combinations that give similar patterns of neural activity in this network (Leonardo, 2005). For example, more than 20 million different combinations of parameter values were analyzed for a model of one of the chains from the lobster stomatogastric ganglion in a study by Prinz et al. (2004). Of these, about 2% yielded dynamics statistically indistinguishable from the three-phase rhythm observed experimentally in this network.

The dynamical degeneracy is related to the fact that various patterns of neural activity can lead to the same behavioral patterns. For example, the study (Leonardo, 2005) describes the brain structures controlling singing of birds. One of the structures is the robust nucleus of the arcopallium (RA) that has a projection on spinal cord neurons controlling the voice muscles. Within one period of a sound pattern, RA neurons pass through a number of spike activity states that are not repeated during this period. At the same time, the song itself contains intervals with a similar sound pattern within one period. Therefore, different spike patterns can cause similar muscle contractions. This degeneracy may be associated with the presence of functionally “neutral (neither useful nor harmful) variability” of neural signals when the brain solves a repeated behavioral task using various neural activity patterns that lead to the same movements (synonymous commands).

Another example of coding in the presence of synonymous commands is coding with sequences of Markov model states, which is described in Abeles et al. (1995), Jones et al. (2007). In these studies, each individual record of neuron spike activity is divided into intervals in which each neuron has an approximately constant spike frequency. During each of these intervals, the neural network remains in a particular state; interstate transitions are described by a Markov model. For a particular sensory stimulus, the set of the states and the sequence in which they appear are repeated with a high precision from trial to trial, while the length of stay in each state is variable. These patterns form a set of synonyms coding for the same stimulus and, probably, are similarly interpreted by the subsequent brain areas. Although synonymous spike patterns can in general lack common structural features (as is often the case for synonyms in a natural language), we cannot exclude the existence of other regular types of dynamical degeneracy.

The structural degeneracy is related to implementation of the same function by different neuronal groups. According to the neural Darwinism theory, this degeneracy is the basis of brain development through life, providing a diversity of structures for natural selection.

## Useful variability and the neural Darwinism theory

Of particular importance is the genuine useful variability, which we will consider in terms of the neural Darwinism theory. The term “neural Darwinism” (selection of neuronal groups) was introduced by G. Edelman in 1987 to explain the course of ontogenetic development of the nervous system, based on the principles of Darwin's natural selection: “Variation and selection within neural populations play key roles in the development and function of the brain” (Edelman, 1993).

Selection of neurons during ontogeny is a competition for sources of oxygen, glucose, and connections with other neurons. The competition among neurons begins with a competition among synapses. Edelman distinguished three stages of neuronal group selection: selection of highly specialized groups in early ontogeny, secondary selection of neural groups through personal experience and an improved efficiency of synaptic connections, and the formation of “reentrant signaling,” which results in integration of a current state with long-term memory traces.

According to the Edelman theory, a newborn perceives the world as a set of disordered chaotic signals, and only acquired experience (not a predetermined instruction) enables classification of these signals, identification of signal combinations perceived as a particular object, and correlation of the signals with pleasant or threatening events. Fixation of individual experience occurs through competitive selection of groups of neurons and synapses, with the selection patterns being similar to the patterns of natural selection in evolving populations.

For example, neurons in the visual cortex are not initially specialized, and each neuron responds to a wide range of signals. These spectra can slightly vary for individual neurons. There are numerous interneuronal synaptic contacts formed more or less randomly. When a visual stimulus (e.g., a dash moving up and right) is presented, all neurons responding to it emit electrical pulses at the same frequency. When the stimulus disappears, the neurons can stay active, but the pulses are no longer correlated. Contacts among synchronously responding neurons are amplified, and the neurons are combined into a group. Later, excitement of one of the neurons from the group induces the activation of other neurons, i.e., the entire group starts to respond to stimuli as a whole. Synapses among neurons whose activity periods are not identical weaken or even disappear.

The formation of these neuronal groups in different cortical areas (visual, auditory, and motor) starts even before birth. Edelman calls the groups composed of neurons with initially disordered contacts the primary repertoire. Groups acquire certain specializations. For example, some groups in the visual cortex respond more to vertical bars, other groups respond to horizontal bars, and still other groups respond to sloped bars. Many groups respond to each signal, with some groups responding better than others. The possibility of the formation of these specialized groups developed during evolution and was genetically preserved. However, the cortex structure in each particular case, i.e., how neurons are combined in groups and what their specialization is, is not predetermined either by a genetic program or by the environment. Only the general organization of cortical areas (visual, auditory, association, etc.) is genetically determined.

According to the theory, the formation of neuronal groups at all stages conforms to the principles of Darwinian selection. However, the selection occurs not in successive generations of individuals but in populations of somatic (nerve) cells connected through synapses. The population approach widely developed by Darwin considers intraspecies variations not as mistakes, but as a prerequisite and source of diversity that is used by natural selection to generate various kinds of organisms. During evolution, mutations, i.e., random events, recombination, and gene drift serve as the major sources of variability; phenotypic functions provide exposure to the environment and selection of the fittest organisms; differential reproduction of phenotypes leads to preservation of selection results in the next generation. According to the Edelman concept, the variability during the ontogenetic brain development arises due to random connections in ensembles of neuronal groups and due to strengthening or weakening of existing synapses; the behavior (initially, in the form of primarily irregular reactions, but then, as categories form, becoming more and more ordered) leads to exploring the environment; adaptive responses, which are repeated more often than others, are accompanied by preservation and strengthening of synapses of the neuronal groups involved in the responses (Edelman, 1993; Seth et al., 2006).

Thus, randomness and variability underlie the formation of new mutations in the genotype and the gene drift, which results in evolution upon fixation of a useful mutation. Randomness also plays a big role in the formation of synapses. One of the possible governing principles is the so-called Peters rule: “According to the “Peters rule”, synaptic contacts occur where dendrites and axons happen to be in apposition. These “potential synapses” are required but not sufficient for an actual synapse formation; and the expected number of connections between two neurons is proportional to the product of their dendritic and axonal trees densities” (Peters et al., 1991; Peters and Payne, 1993).

## Experimental evidence supporting the existence of useful variability

Variability of neuronal responses can be one of the key drivers of neuronal group selection. This hypothesis is supported by recent studies on the auditory cortex of rats. Takahashi et al. (2013) demonstrated that functional maps and plasticity of the auditory cortex in rats correlated with variability of neuronal responses, namely with the variability of mutual information of the neural activity and the stimulus as well as with the spike frequency. The degree of response variability in functional units of computation (tonotopic columns and auditory fields) is likely co-modulated with the representational area in accordance with training and experience. In other words, large representational areas promote the formation of a heterogeneous population of neurons that emit various responses to stimuli. These results indicate that the functional map plays an important role in the implementation of the Darwinian principles in cortical computations. The model proposed by the authors can account for functional roles as well as some specific features of plasticity of cortical maps (Takahashi et al., 2013).

One of the important conclusions of the neural Darwinism theory is that models based on it are capable of self-learning. A line of Darwin computer models was developed based on the neural Darwinism theory. For example, two forms of learning were implemented in the Darwin VII model: perceptual categorization based on plasticity of cognitive neurons and reflex conditioning controlled by motor system plasticity upon action of taste reinforcement (Sokolov and Nezlina, 2005).

Ölveczky et al. (2011) studied neuronal activity of the bird RA area responsible for the song generation. It was found that the variability of neuronal responses in this area decreased during the learning to sing, and adult birds had highly stereotypical spike patterns. Experiments involving the inactivation of RA presynaptic regions identified an area generating this variability—the lateral magnocellular nucleus of the anterior nidopallium (LMAN). The presence of this area suggests that response variability during learning may be specifically generated by the brain, which would likely be evidence that such variability is useful for neuroplasticity processes (Ölveczky et al., 2011). It is particularly interesting if there are analogs of the LMAN area in primates. According to one view, an analog is the basal ganglia-thalamocortical loops (Ölveczky et al., 2011). Another suggested functional analog is the premotor cortex (PMC) (Jarvis, 2004). This assumption is supported by the fact that a temporary reduction in the premotor cortex activity leads to a decrease in the learning ability (Mochizuki et al., 2005). Similarly, damage to the premotor cortex in stroke reduces capabilities for planning of movements and motor learning (Chang et al., 2010).

## Conclusion

The phenomenon of variability of neuronal responses has many facets concerning its causes and functional significance for cognitive activity. To adequately account for variability in interpretation of experimental results, the identification of its functional meaning is required. For example, false variability determined by unknown regular factors may potentially be reduced through additional control of experimental parameters; genuine harmful variability can be modeled as random noise; genuine neutral variability indicates the presence of synonymous commands due to functional degeneracy, which should be taken into account in the decoding of information transmitted by neuronal signals. Finally, the most important, genuine useful, variability should be interpreted as purposefully generated and regulated fluctuations of the neural activity that likely enable involvement of various neuronal groups in different trials, subjected to natural selection to fix the most effective responses. This genuine useful variability may serve as the basis for neuroplasticity and learning.

## Author contributions

AC, DS—review literature, prepare manuscript, generate the ideas; MP—overall coordination, discussion of the results.

### Conflict of interest statement

The authors declare that the research was conducted in the absence of any commercial or financial relationships that could be construed as a potential conflict of interest.
